# SET7/9 exhibits sigmoidal kinetics on nucleosomes, hyperbolic kinetics on histones by an ordered sequential mechanism, and methylates lysine and arginine

**DOI:** 10.1016/j.jbc.2025.110639

**Published:** 2025-08-28

**Authors:** Olufola O. Ige, Thordur Hendrickson-Rebizant, Wenxia Luo, Phinehas Cheung, Ying Lao, Rene P. Zahedi, James R. Davie, Ted M. Lakowski

**Affiliations:** 1 Pharmaceutical Analysis Laboratory, College of Pharmacy, University of Manitoba, Winnipeg, Manitoba, Canada; 2 Paul Albrechtsen Research Institute, CancerCare Manitoba, Winnipeg, Manitoba, Canada; 3 Manitoba Centre for Proteomics and Systems Biology, Winnipeg, Manitoba, Canada; 4 Department of Internal Medicine, Winnipeg, Manitoba, Canada; 5 Department of Biochemistry and Medical Genetics, University of Manitoba, Winnipeg, Manitoba, Canada

**Keywords:** epigenetics, enzyme kinetics, hyperbolic kinetics, SET7/9, lysine methyltransferase, liquid chromatography tandem mass spectrometry (LC-MS/MS), proteomics, histone H2BK120 ubiquitinated nucleosome core particles, sigmoidal kinetics, histone lysine methylation, nucleosome core particles

## Abstract

SET7/9 (SETD7) is a SET domain protein lysine methyltransferase (PKMT). We characterized its activity using a mass spectrometry (MS) assay, showing that it follows an ordered sequential enzyme kinetic mechanism where SAM is the first substrate to bind, followed by histone H3, and mono-methylated histone H3 is the first product to dissociate, followed by SAH. Full-length histones H2A, H2B, and H4 are also substrates for SET7/9. We found that SET7/9 methylates nucleosome core particles (NCP), histone H2BK120 ubiquitinated nucleosome core particles (Ub-NCP) as well as histone octamers, exhibiting sigmoidal kinetics, and suggesting an allosteric interaction that was not observed with free histones, which follow hyperbolic (Michaelis–Menten) kinetics. Using low (25 nM) SET7/9, we only detect monomethyl-lysine with all substrates except Ub-NCP, which produces mono- and di-methyllysine at equal rates. Proteomic analysis shows that SET7/9 catalyzes multiple sites and types of, methylation on histones, depending on if they are free histones, or within an octamer. Free histone H3 is methylated at multiple N-terminal sites (including H3K4) that are not methylated in the octamer. Some C-terminal methylation sites were discovered that can also be ubiquitinated including H2BK120. Since lysine methylation and ubiquitination are mutually exclusive such methylation by SET7/9 may serve to prevent ubiquitination. At high enzyme and substrate concentrations and extended incubation times, we show that SET7/9 can catalyze the formation of dimethyl- and trimethyllysine on H2B, H3 and histone octamers and, most remarkably, the formation of monomethyl- and dimethylarginine on histone H3 and within octamers.

The protein lysine methyltransferases (PKMTs) are a family of enzymes requiring two substrates and producing two products following a Bi-substrate and Bi-product (Bi-Bi) enzymatic mechanism. Accordingly, they transfer methyl groups from the substrate S-adenosyl-L-methionine (SAM) to the ε-amino group of lysine residues on protein substrates, producing the products S-adenosyl-L-homocysteine (SAH), and methyllysine residues (1). Perhaps the most famous PKMT substrates are histones where their methylation influences gene expression through epigenetic processes.

Depending on the PKMT and protein substrate up to three methyl-groups can be transferred forming mono-, di- and trimethyllysine (Kme1, Kme2, and Kme3, respectively) (Fig. 1) (2). Methylation of lysine does not affect its charge but may interfere with hydrogen bonding. Methyl-lysines bind to what are known as reader domains within effector proteins like transcription factors, co-activators, co-repressors, or other proteins that often change gene expression. Histone methyl-lysine recognition by reader domains is context-dependent as it can be based on the number of methyl-groups on the target lysine, the surrounding amino-acid sequence, and their potential post-translational modifications (3).

**Figure 1:**
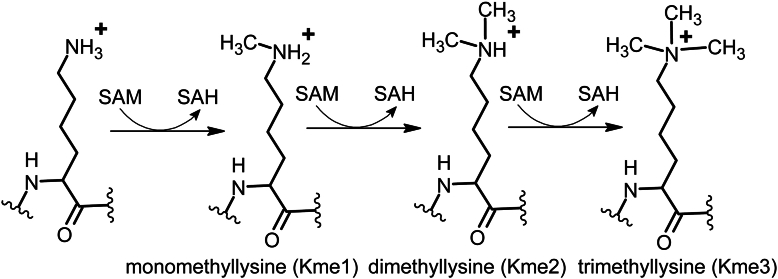
**Reactions catalyzed by protein lysine methyltransferases**. PKMT enzymes can add up to three methyl groups to lysine residues on their protein substrates.

Histone lysine methylation can be associated with heterochromatin or euchromatin and, low and actively transcribed regions, respectively. Generally, methylation of histone H3K4, H3K36, and H3K79 are associated with active transcription, while methylation on histone H3K9, H3K27 and H4K20 are associated with decreasing transcription (4). However, the context of other histone modifications can influence the overall effect on expression.

PKMTs typically methylate particular histone N-terminal tail residues, and a maximal number of methyl-groups are added. In addition to histone N-terminal tails, core and C-terminal lysine residues can also be methylated or be the site of other modifications such as acetylation and ubiquitination but not at the same time. Since such modifications cannot simultaneously be on the same lysine residue, they are said to be mutually exclusive.

SET7/9 is a SET domain PKMT, so called because it contains a SET methylation domain (5). It was initially given the names SET7 (6) and SET9 (7). Although it is now formally known as SETD7, the name SET7/9 is more commonly used and will be used throughout this manuscript. SET7/9 is known for catalyzing mono-methylation of histone H3K4 (H3K4me1), where it is thought to play a role in active enhancers (8), but with the modification being added prior to nucleosome assembly, since SET7/9 was previously not known to methylate nucleosomes (7). SET7/9 has demonstrated limited ability to mono-methylate peptides representing the N-terminal tails of histones H2A and H2B (9, 10) and histone H1 (11). Although SET7/9 methylates non-histone proteins the focus of this study is on the histone substrates of SET7/9.

SET7/9 binds the target lysine residue through a channel lined with conserved tyrosine residues (12). The hydroxyl groups of such conserved residues Y245 and Y305, form hydrogen bonds with the target lysine residue, and this dictates the maximal number of methyl-groups the enzyme can add. For example, wild-type SET7/9 is thought to only catalyze histone H3K4me1, but the mutation Y245A produces histone H3K4me1 and di-methylation (H3K4me2) (12), whereas Y305F produces H3K4me2 and small amounts of tri-methylation (H3K4me3) (6).

Here we investigated the methylation activity of SET7/9 on free histone and nucleosome substrates to determine enzymatic mechanism and to see if it can methylate nucleosomes. A validated liquid chromatography tandem mass spectrometry (LC-MS/MS) assay was used to quantify the modified amino acids Kme1, Kme2, and Kme3 as well as mono-, and asymmetric and symmetric dimethylarginine (Rme1, Rme2a, Rme2s) derived from quantitatively hydrolyzed reactions containing SET7/9 and free histone and nucleosome substrates. We show that SET7/9 follows an ordered sequential Bi-Bi enzymatic mechanism where SAM is the first substrate to bind, followed by histone H3, and mono-methylated histone H3 is the first product to dissociate, followed by SAH. Full-length free histones H2A, H2B and H4 are also substrates for SET7/9. For the first time, we find that SET7/9 methylates, histone octamers, nucleosome core particles (NCP), and H2BK120 mono-ubiquitinated nucleosome core particles (Ub-NCP) following sigmoidal enzyme kinetics and suggesting an allosteric interaction that was not observed with free histones which follow typical hyperbolic (Michaelis–Menten) kinetics. Based on previous studies, we suggest that Membrane Occupation and Recognition Nexus MORN repeats of SET7/9 are the allosteric site for interactions with histone H3 in the context of a nucleosome. We conducted the first proteomic analysis of the methylation of histones by SET7/9 discovering multiple sites, and types of methylation that differ depending on if the histones are free or within an octamer. Free histone H3 is methylated at multiple N-terminal sites but almost none of these sites, including the active mark histone H3K4, are methylated in the histone octamer. Many of the sites of histone lysine methylation are also sites of mono-ubiquitination (Ub), including H2BK120, and these modifications are mutually exclusive. We theorize that the concerted effect of SET7/9 on nucleosomes may be to decrease histone H3K4me1 and thereby reduce the activity of active enhancers. With high enzyme and substrate concentrations and extended incubation SET7/9 catalyzes the formation of Kme2 and Kme3 on H2B, H3, and histone octamers and, most remarkably, the formation of Rme1 and Rme2a on histone H3 and histone octamers.

## Results

The method we used to measure the enzymatic activity of SET7/9 was adapted from our previous validated method except we utilized a volatile buffer (NH_4_)HCO_3_ to reduce background and ion suppression, and incorporated stable isotope labeled ^13^C6 ^15^N2 lysine as an internal standard to correct for loss during sample processing and injection (13, 14, 15). The details are briefly summarized in the Materials and Methods and supplementary materials.

At high (25 μM) and low (5 μM) concentrations of histone H3, the rate of methylation (represented by rate of production of Kme1) by SET7/9 (25 nM) is linear with respect to time up to at least 60 min at 30 °C (Fig. S1). The rate of production of Kme1 is linear with respect to enzyme concentration from 3.125 to 200 nM SET7/9 (Fig. S2). The pH optimum was identified as 9 (Fig. S3), and DTT did not influence enzyme rate (Fig. S4). Therefore, all subsequent kinetic reactions were carried out for 20 min with 25 nM SET7/9 in a buffer with 100 mM (NH_4_)HCO_3_ at pH 9, and 200 nM ^13^C6 ^15^N2 lysine internal standard at 30 °C, unless otherwise stated. Previous studies apparently showed SET7/9 catalysed methylation of itself (AKA auto-methylation) but this was only in the presence of the transcription factor Yin Yang 1. Auto-methylation experiments with only SET7/9 and SAM were never performed (16). Notably, our substrate control assays which contained SET7/9, and SAM but no histone showed no detectable methylation activity suggesting that SET7/9 does not auto-methylate (Fig. S5).

### SET7/9 follows a sequential mechanism

**Figure 2:**
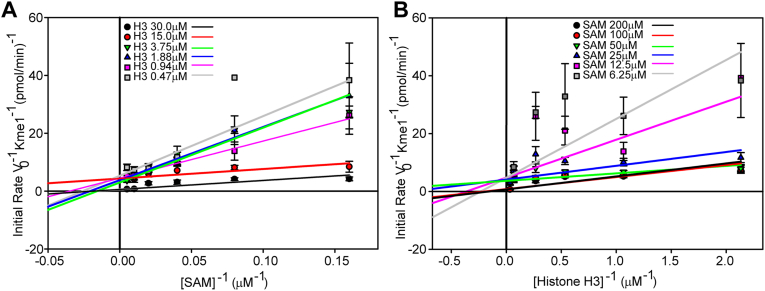
**Sequential Bi-Bi substrate enzymatic mechanism for SET7/9**. Reciprocal plots of the initial velocity (v_o_) of SET7/9 with the substrates, full-length histone H3 and SAM. The concentration of SAM was varied with fixed histone H3 concentrations from 0.47 to 30 μM (*A*). These data were replotted where the concentration of histone H3 was varied with fixed SAM concentrations from 6.25 to 200 μM (*B*). The initial velocity was measured by quantifying the amount of Kme1 produced over 20 min. Each point is the mean and SD of three reactions. The kinetic parameters derived from these graphs and the replots in Figure S8, are shown in Table 1.

The enzymatic mechanism of an enzyme determines how substrates bind to that enzyme and allows determination of its enzymatic parameters. To start determination of the enzymatic mechanism of SET7/9, we incubated the enzyme with a range of increasing concentrations of histone H3 and SAM. First, we estimated the V_Max_ and K_M_ values to determine these concentration ranges by varying each substrate in the presence of a fixed excess of the other. Based on these initial values (apparent values) the concentration ranges for SAM and histone H3 were selected for further experimentation. When histone H3 was varied in the presence of several constant SAM concentrations, the double reciprocal plots exhibit increasing slopes with decreasing SAM concentration, producing a pattern of lines intersecting to the left of the y-axis, above the x-axis (Fig. 2). The pattern of lines suggests that the substrates bind sequentially, therefore following a sequential Bi-Bi substrate binding mechanism, rather than a double displacement Bi-Bi substrate enzymatic mechanism where the methyl-group from SAM would transiently attach to the enzyme before being transferred to the substrate. Such a sequential mechanism is consistent with most (17), but not all SAM dependent methyltransferases (18).

### Product inhibition studies

**Figure 3:**
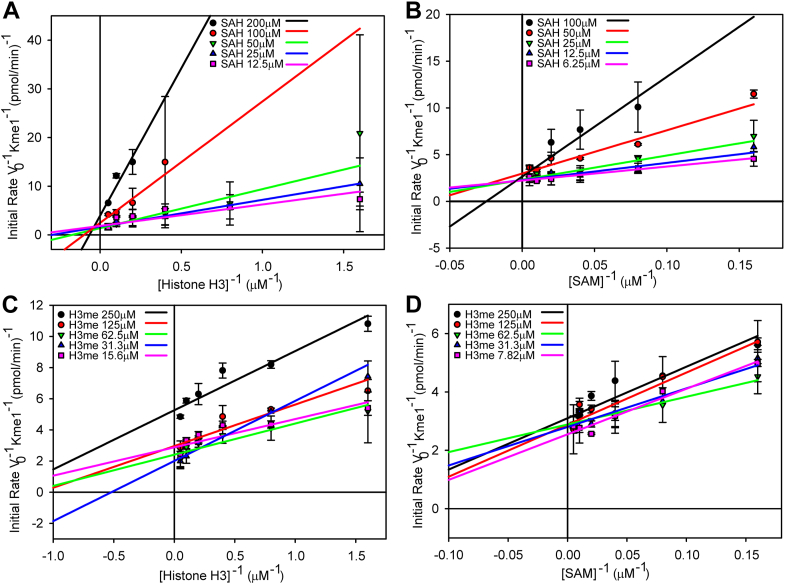
**SET7/9 follows an ordered sequential Bi-Bi enzymatic mechanism**. Reciprocal plots of the initial velocity (v_o_) of SET7/9 with the substrates, full-length histone H3 and SAM, and the product inhibitors SAH and histone H3K4me1MLA (H3me). Plotted are fixed concentrations of the product inhibitor SAH with variable histone H3 concentrations and a fixed SAM concentration of 30 μM (*A*) or variable SAM and a fixed histone H3 concentration of 4 μM (*B*). Shown below them are fixed concentrations of the product inhibitor histone H3K4me1MLA with variable histone H3 concentrations and a fixed SAM concentration of 30 μM (*C*) or variable SAM and a fixed histone H3 concentration of 4 μM (*D*). The initial velocity was measured by quantifying the amount of Kme1 produced over 20 min. Each point is the mean and SD of two reactions. The replots of the slopes and intercepts from these graphs are shown in Figure S9. The product inhibitor constants and their mathematical descriptions are shown in Table S2 and supplemental equations Eqs S1–S7, respectively.

**Figure 4:**
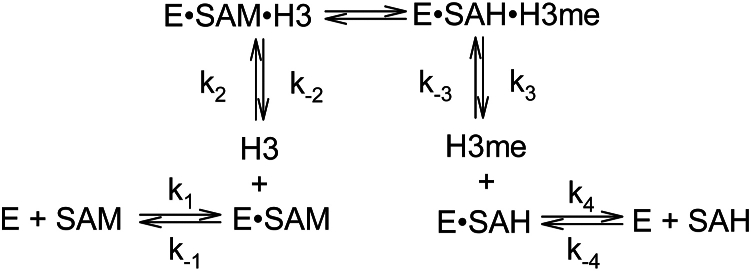
**The kinetic mechanism of SET7/9.** Shown is the ordered sequential Bi-Bi enzymatic mechanism of SET7/9 (represented as E) where SAM is the first substrate to bind, followed by histone H3 (H3), and the first product to dissociate is monomethylated histone H3 (H3me), followed by SAH. The microscopic forward k_n_ and reverse k_-n_ rate constants are shown for each step n of the reaction. With respect to equations 1 through 7, SAM is substrate A, and histone H3 is substrate B. For supplemental equations 1 through 7 (Eqs S1–S7) and Table S2, monomethylated histone H3 is product P, and SAH is product Q.

The data in Figure 2 suggest a sequential mechanism of substrate binding to SET7/9 but cannot determine if the substrate binding and product release has to follow a strict order where one substrate must bind first before the other, and one product must dissociate first before the other (an ordered sequential Bi-Bi mechanism), or if either substrate can randomly bind first and either product dissociate first (a random sequential Bi-Bi mechanism). Both mechanisms form a stable ternary complex composed of both substrates and the enzyme, whereas a special case of the ordered sequential Bi-Bi mechanism, known as the Theorell Chance mechanism, does not. To determine which of these mechanisms SET7/9 follows, product inhibition assays must be performed where the products of the reaction, called product inhibitors, are added to the reaction to determine what type of inhibition they produce, since this informs us about how the substrates bind to the enzyme. We used constant and variable substrates, SAM and histone H3, and increasing concentrations of the product inhibitors histone H3Kme1 methylated lysine analog (MLA) (H3K4me1MLA) or SAH. Preliminary measurements of the inhibition of SET7/9 by histone H3K4me1MLA (Fig. S6) and SAH (Fig. S7) were performed to find an appropriate concentration range for each product inhibitor. Figure 3*A* shows the inhibition of SET7/9 by SAH with a constant non-saturating concentration of SAM and variable histone H3 concentrations. The curves appear to converge to the left of the y-axis, indicating mixed inhibition. In contrast, Figure 3*B* shows the inhibition of SET7/9 by SAH with a constant non-saturating concentration of histone H3 and variable SAM, where the curves appear to converge on the y-axis. This suggests competitive inhibition by SAH, where SAM and SAH compete for the SAM/SAH binding site. Such inhibition suggests an ordered sequential Bi-Bi mechanism, in which SAM is the first substrate to bind and SAH is the last product to dissociate from SET7/9. These data also argue against a random sequential Bi-Bi mechanism, which would yield mixed inhibition. Mixed inhibition of SET7/9 is observed with increasing concentrations of histone H3K4me1MLA in Figure 3*C*, where histone H3 is varied with fixed SAM. Such inhibition is indicative of an ordered sequential Bi-Bi mechanism, but not a Theorell-Chance mechanism, which would exhibit competitive inhibition. Figure 3*D* shows the inhibition of SET7/9 by H3K4me1MLA, where the SAM concentration varies with fixed histone H3. Most of the curves appear to converge to the left of the y-axis, suggesting a mixed type of inhibition and an ordered sequential Bi-Bi mechanism (17). Therefore, we conclude that SET7/9 follows an ordered sequential Bi-Bi mechanism, where SAM binds to the enzyme first, followed by histone H3, and the product methylated histone H3 dissociates from the enzyme first, followed by SAH (Fig. 4). These substrates and products are conventionally referred to as substrates A and B, and products P and Q, respectively.

The initial rate (v_o_) of the reaction for an ordered sequential Bi-Bi mechanism can be described using Equation 1 and its reciprocal forms, Equation 2, where the first substrate A is varied and the second substrate B fixed, and Equation 3, where B is varied and A fixed. K_SA_ is the equilibrium dissociation constant for the first substrate to bind to SET7/9, K_MA_ and K_MB_ are the Michaelis constants for the substrates A and B, respectively, V_Max_ is the maximum rate of the reaction, and [A] and [B] are the concentrations of the substrates. The turnover number or k_cat_ is defined according to Equation 4 (19).

(1) $$v_{0}=\frac{V_{\mathrm{Max}}\left[A\right]\left[B\right]}{K_{SA}K_{MB}+K_{MB}\left[A\right]+K_{MA}\left[B\right]+\left[A\right]\left[B\right]}$$

(2) $$\frac{1}{v_{0}}=\frac{K_{MA}}{V_{\mathrm{Max}}}\left(\frac{K_{SA}K_{MB}}{\left[B\right]K_{MA}}+1\right)\frac{1}{\left[A\right]}+\frac{1}{V_{\mathrm{Max}}}\left(\frac{K_{MB}}{\left[B\right]}+1\right)$$

(3) $$\frac{1}{v_{0}}=\frac{K_{MB}}{V_{\mathrm{Max}}}\left(\frac{K_{SA}}{\left[A\right]}+1\right)\frac{1}{\left[B\right]}+\frac{1}{V_{\mathrm{Max}}}\left(\frac{K_{MA}}{\left[A\right]}+1\right)$$

(4) $$k_{cat}=\frac{V_{\mathrm{Max}}}{{\left[E\right]}_{T}}=\frac{k_{3}k_{4}}{k_{3}+k_{4}}$$

Knowing that it follows an ordered sequential Bi-Bi mechanism, the kinetic parameters for SET7/9, V_Max_, K_SA_, K_MA_, K_MB_, and K_i_ (Table 1) were determined by replotting the slopes and intercepts from the double reciprocal plots from Figure 2 in Figure S8, and by replotting the slopes and intercepts from product inhibitor plots from Figure 3 in Figure S9. The turnover numbers, k_cat_ values, were calculated using Equation 4, and the specificity constants (k_cat_/K_M_) were calculated using the derived parameters. As expected, the V_max_ for both histone H3 and SAM is approximately the same. However, the K_M_ and k_cat_/K_M_ for SAM are almost 9-fold and 8-fold higher than the corresponding parameters for histone H3. Since the reaction follows an ordered sequential Bi-Bi mechanism and we are not using saturating concentrations of either substrate, the K_i_ for SAH (Table 1, defined as K_iQ_ in the supplemental equation Equation S5) is the only true K_i_ derived from the product inhibitor studies (17). A full description of the product inhibitor slope and intercept inhibition constant equations (19), and their values, is available in the Supplementary material (Equations S1–S7, and Table S2).

**Table 1:** Kinetic parameters for SET7/9 substrates

Compound	Type	K_M_ (μM)	K_SA_ (μM)	K_i_ (μM)	V_Max_ (pmol/min)	k_cat_ (s^−1^)x10^−3^	k_cat_/K_M_ (M^−1^s^−1^)
SAM	Substrate	11.99 ± 3.17	37.86 ± 19.8		0.5357 ± 0.128	5.953 ± 1.425	498.6 ± 32.2
Histone H3	Substrate	1.358 ± 0.219			0.4719 ± 0.0482	5.243 ± 0.535	3971 ± 1050
SAH	Product			12.14 ± 3.11			

### Histone and nucleosome substrate profiling

**Figure 5:**
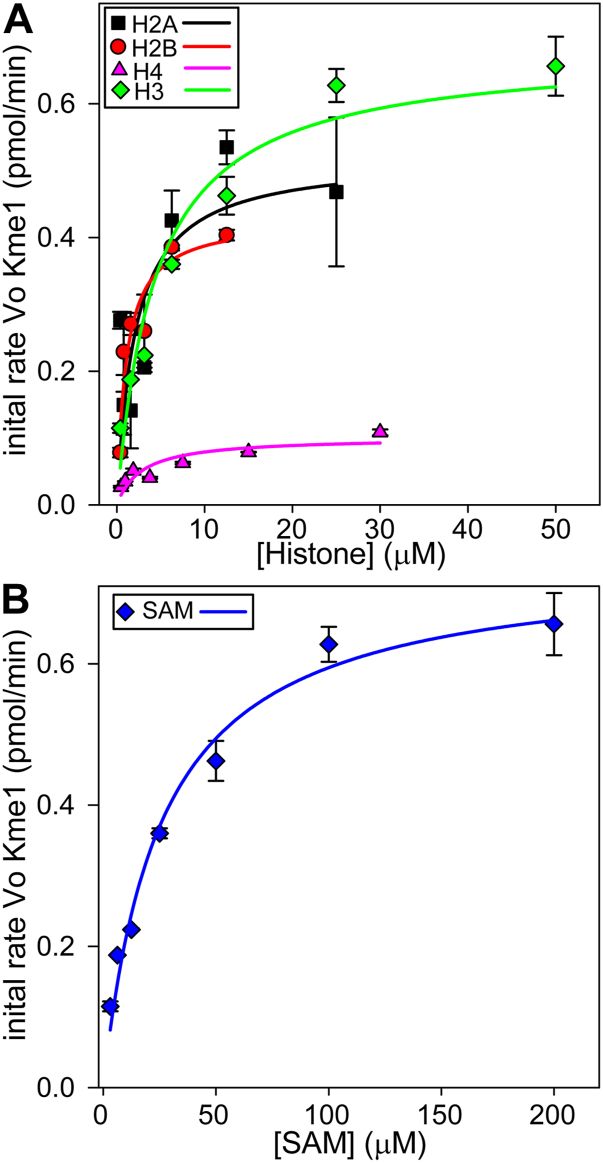
**Substrate profiling for SET7/9 with free histones and SAM**. The initial rate of production of Kme1 by SET7/9 with varying concentrations of histone H3, H2A, H2B, or H4 with a fixed SAM concentration of 200 μM (*A*) or varying SAM with a fixed histone H3 concentration of 30 μM (*B*). The initial velocity was measured by quantifying the amount of Kme1 produced over 20 min. Each point is the mean and SD of two reactions. The curves were fit to Equation 5 (*A*)and Equation 6 (*B*), and the apparent Michaelis–Menten kinetic parameters are shown in Table 2.

The activity of SET7/9 against free histones, histone octamers, NCP, and Ub-NCP was compared using apparent kinetic parameters for an unbiased comparison among substrates. Plots of initial reaction rates as measured by the amount of Kme1 produced per unit time with increasing free histones H3, H2A, H2B, and H4 and fixed saturating SAM are shown in Figure 5. To calculate apparent kinetic parameters, we used saturating concentrations of substrate B (histones), where Equation 2 reduces to Equation 5, and similarly used saturating concentrations of substrate A (SAM), where Equation 3 reduces to Equation 6 (*i.e.* one substrate Michaelis–Menten kinetics, AKA hyperbolic kinetics). The rate data for SET7/9 against histones H3, H2A, H2B, and H4 were fit to Equations 5 and 6 to derive apparent kinetic parameters (Table 2). The apparent V_Max_ (V_Max App_) for histone H3 and SAM are 20 to 30% higher than the actual values in Table 1, and the apparent K_M_ (K_M__App_) for histone H3 and SAM are 3- and 1.7-fold higher, respectively, than the actual values determined by our kinetic analysis (Table 1). Using the apparent specificity constant ((k_cat_/K_M_)_App_), the preference of SET7/9 for free histones is H2B > H2A > H3>>H4. Contrary to others, we found that SET7/9 does methylate histone H4 but with V_Max App_ values less than ⅙ to one-fourth of that for histones H3, H2A, and H2B.

(5) $$v_{0\;App}=\frac{V_{\mathrm{Max}\;App}\left[A\right]}{K_{MA\;App}+\left[A\right]}$$

(6) $$v_{0\;App}=\frac{V_{\mathrm{Max}\;App}\left[B\right]}{K_{MB\;App}+\left[B\right]}$$

**Table 2:** Apparent Michaelis–Menten kinetic parameters for SET7/9 with histones

Variable substrate	Saturating Substate	K_M__App_ (μM)	V_Max__App_ (pmol/min)	k_cat__App_ (s^−1^)x10^−3^	(k_cat_/K_M_)_App_ (M^−1^s^−1^)
SAM	Histone H3	20.49 ± 1.76	0.6862 ± 0.0302	7.623 ± 0.335	373.1 ± 37.8
Histone H3	SAM	4.383 ± 0.416	0.6525 ± 0.0460	7.250 ± 0.511	1656 ± 40.5
Histone H2A	SAM	1.989 ± 0.209	0.5113 ± 0.0547	5.682 ± 0.607	2857 ± 5.057
Histone H2B	SAM	1.067 ± 0.255	0.4286 ± 0.0239	4.762 ± 0.266	4565 ± 842
Histone H4	SAM	2.791 ± 0.0252	0.1013 ± 0.00156	1.126 ± 0.0173	403.4 ± 9.84

**Figure 6:**
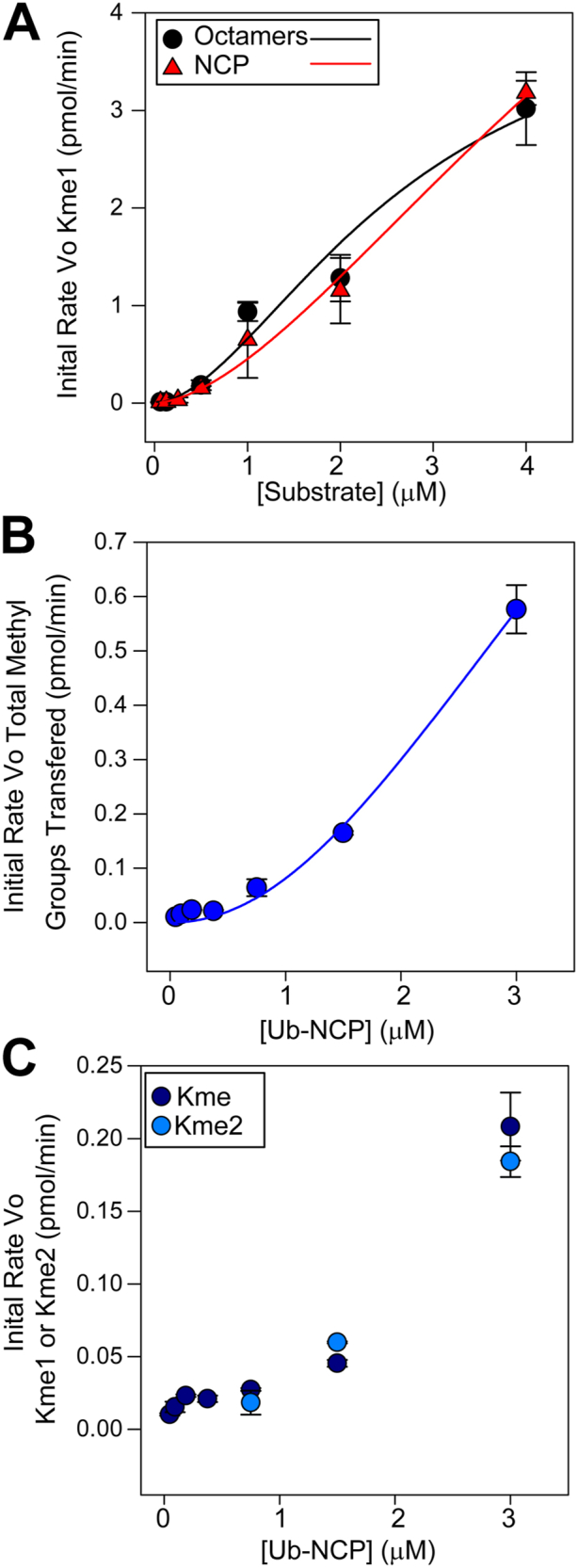
**Substrate profiling for SET7/9 with histone octamers, NCP, and Ub-NCP**. The initial rate of production of Kme1) in pmol per min by SET7/9 with varying concentrations of histone octamers or NCP with a fixed SAM concentration of 200 μM (*A*). The initial rate of addition of total methyl groups as measured by the weighted sum of Kme1 and Kme2 (Kme1+2Kme2) in pmol of methyl-groups per min by SET7/9 with a fixed SAM concentration of 200 μM (*B*). The initial rate of production of Kme1 and Kme2 in pmol per min by SET7/9 with a fixed SAM concentration of 200 μM. Each point is the mean and S.D. of two repeats, except for 6A NCP, which is the mean and SD of four repeats. The curves in A and B were fit to Equation 7and the kinetic parameters are shown in Table 3.

Since previous studies showed that SET7/9 cannot methylate NCP (20, 21, 22, 23, 24), we measured the activity of SET7/9 against NCP, Ub-NCP, and histone octamers. Interestingly, SET7/9 methylated histone octamers, NCP, and Ub-NCP exhibited sigmoidal (*i.e.* non-Michaelis–Menten) kinetics when measuring initial rate with increasing octamers or NCP (Fig. 6A) or Ub-NCP (Fig. 6, *B* and *C*) with saturating SAM. Such sigmoidal curves suggest an allosteric interaction between NCP, Ub-NCP, histone octamers, and SET7/9. We could not saturate the reaction since we could not reach concentrations higher than 4 μM for NCP and histone octamers and 3 μM for Ub-NCP. Nevertheless, even at these low substrate concentrations, the sigmoid shape is evident, and the V_Max_ _App_ was at least 6.2 to 9-fold higher for NCP and histone octamers than the V_Max_ _App_ for any of the free histones. Since the curves were sigmoidal, the data from Figure 6, *A* and *B* were fit to the Hill equation (Equation 7), and the apparent kinetic parameters are listed in Table 3. Interestingly, Ub-NCP is the only substrate that produces Kme1 and Kme2 at equal rates (Fig. 6*C*). All other SET7/9 substrates only produce Kme1 under these conditions.

(7) $$v_{0\;App}=\frac{V_{MaxApp}{\left[B\right]}^{n_{H}}}{K_{B\;App}^{,n_{H}}+{\left[B\right]}^{n_{H}}}$$

**Table 3:** Apparent sigmoidal kinetic parameters for SET7/9 NCP and histone octamers

Variable substrate	Saturating Substate	K^’^_MB__App_ (μM)	V_Max__App_ (pmol/min)	n_H_	k_cat__App_ (s^−1^) x10^−3^	(k_cat_/K^'^_MB_)_App_ (M^−1^s^−-1^)
NCP	SAM	3.607 ± 0.354	5.878 ± 0.161	2.062 ± 0.438	65.31 ± 1.78	18,233 ± 1800
Octamers	SAM	2.687 ± 0.0976	4.434 ± 0.561	1.748 ± 0.00712	49.26 ± 6.22	18,302 ± 1650
Ub-NCP	SAM	3.0214 ± 0.334	1.176 ± 0.242	2.418 ± 0.0187	13.07 ± 2.69	4302 ± 414

### Proteomic studies

**Figure 7:**
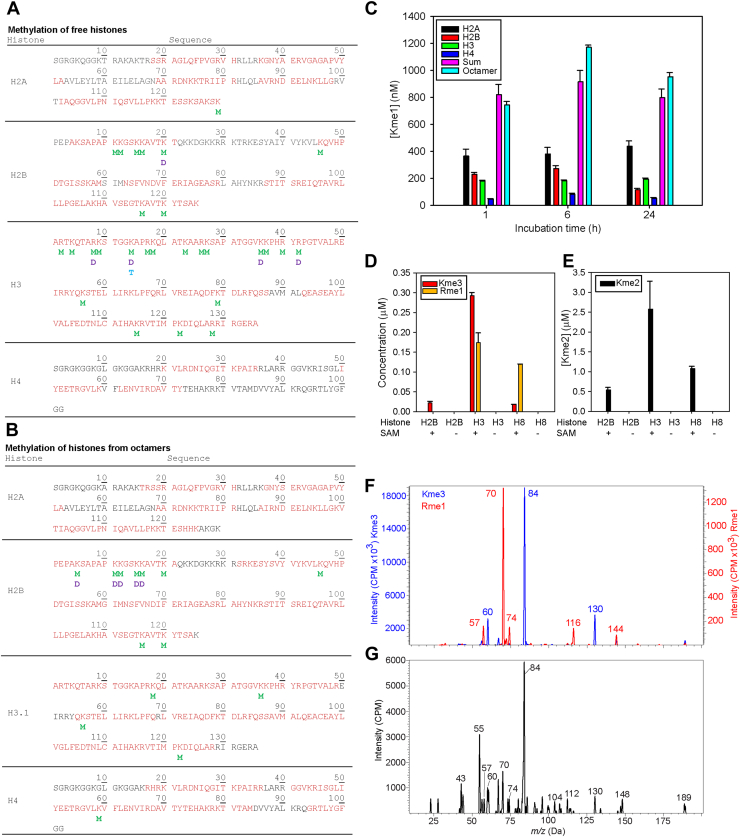
**Analysis of sites and types of methylation on free histones H2A, H2B, H3, and 4H and histone octamers.** The sites of methylation on free *Xenopus laevis* histones H2A, H2B, H3, and H4 (*A*), where M (*green*), D (*purple*), and T (*cyan*), are mono-di and tri-methylation, respectively. Mono- and di-methylation of arginine can be detected on histone H3. The sites of methylation on human histones H2A, H2B, H3, and H4 as part of histone octamers (*B*), where M (*green*), and D (*purple*), are mono-di-methylation, respectively. The peptide sequence coverage is shown in red and the sequence numbering above each line. Methylation of free histones H2A, H2B, H3, and H4 and histone octamers (*C*) at 25 nM SET7/9 and 500 nM of substrate. “Sum” is the sum of methylation of free histones H2A, H2B, H3, and H4. Exhaustive methylation of free histones H2A and H3 and histone octamers (H8) with SET7/9 0.5 μM and SAM 500 μM. Shown is the concentration of Kme3 and Rme1 (*D*) and Kme2 (*E*). Product ion spectra of standards Kme3 (*blue*) and Rme1 (*red*) (*F*). The product ion spectra of a hydrolyzed reaction of histone H3 SET7/9 0.5 μM and SAM 500 μM, recording a precursor ion 189 *m/z* which corresponds to both Kme3 and Rme1 (*G*). This confirms the presence of Kme3 and Rme1 on histone H3. Ion intensity is in counts per minute (CPM) and mass as mass/charge (*m/z*).

H3K4me1 has been assumed to be the only or primary histone product of SET7/9. However, there is some evidence that SET7/9 can methylate histone H2A and H2B tail peptides (9, 10), and the C-terminal residues of the linker histone H1.4(11). Therefore, we performed a proteomic analysis of the methylation of full-length free *Xenopus laevis* histones H3, H2A, H2B, and H4 (Fig. 7*A*) as well as human histone octamers (Fig. 7*B*) to determine all sites of methylation catalyzed by SET7/9. At the relatively low concentrations of histone substrates and SET7/9 in our kinetic studies, and irrespective of incubation time, we could not detect any methylation using proteomics methods. Moreover, under these conditions, our assay could detect only Kme1 in all histones and histone octamers (Fig. 7*C*). To detect methylation and to get sufficient histone sequence coverage, we were forced to use substantially higher SAM and SET7/9 concentrations, in addition to longer incubation times. Under these conditions, SET7/9 catalyzed multiple sites of methylation on free histones, and for the first time, we show that the enzyme can catalyze the formation of Kme3 as well as methylated arginine species (Fig. 7*A*). SET7/9 catalyzed broad-spectrum methylation of free histone H2B and H3, including mono- and di-methylation of H2BK20, and H3K14, H3K36, as well as trimethylation of H3K14. The human histone octamers exhibited different sequence coverage; however, where sequence coverage was congruent, there were many sites of differential methylation, including the lack of methylation on histone H3K4, H3K9, and H3K14 within octamers.

Most surprising is the finding of both mono and di-methylation of arginine on free histone H3R2, H3R8, H3R17, H3R35, and H3R42. We independently confirmed our proteomics studies showing the production of the methylated amino acids Kme2, Kme3, and Rme1 and Rme2a produced from reactions with SET7/9, free histones, and histone octamers using analytical LC-MS/MS, which is different from the proteomic methods used in Figure 7, *A* and *B*. Reactions were prepared similar to the proteomic study with high SAM, enzyme concentrations and extended incubation times but at the end of the incubation, the reactions were hydrolyzed into component amino acids. We quantified Kme1, Kme2, and Kme3, as well as Rme1, primarily on free histone H3 but also to some extent on octamers and free histone H2A (Fig. 7, *D* and *E*). We did not detect any Rme2s but could detect Rme2a; however, it was below our low limit of quantification (LLOQ), so we could not quantify it.

As a third independent method of confirmation that SET7/9 can catalyze the formation of Kme3 and Rme1, we recorded characteristic fragment ion masses derived from product ion mass spectra of Kme3 and Rme1 using analytical LC-MS/MS. Reactions were prepared similar to the proteomic study, but were hydrolyzed into component amino acids to record product ion spectra for Kme3 and Rme1. We recorded the product ion mass spectra of standard pure Kme3 and Rme1 (Fig. 7*F*) and compared these to the hydrolyzed products of reactions with SET7/9, histone H3, and SAM (Fig. 7*G*). Both Kme3 and Rme1 have similar precursor ion mass (189 *m/z*), so the product ion spectra of the hydrolyzed products of the reactions with SET7/9, histone H3, and SAM would be expected to have characteristic product ion masses from both Kme3 and Rme1. Indeed, we found characteristic product ion masses 130, 84, and 60 *m/z* corresponding to Kme3 and 74, 70, and 57 *m/z* corresponding to Rme1, confirming the presence of Kme3 and Rme1 in our reaction samples. As noted, we could detect Rme2a, but the intensity was too low to produce a product ion spectrum. It is important to note that the product ion mass spectra of acid hydrolyzed reactions in Figure 7, *F* and *G* represents a distinct and independent confirmation of the production of Kme3 and Rme1 by SET7/9 from the proteomic studies in Figure 7, *A* and *B*.

## Discussion

### SET7/9 mechanism and product inhibition studies

Protein lysine or arginine methyltransferases typically fall into one of two enzymatic mechanisms: ordered sequential Bi Bi or random sequential Bi Bi. We find that SET7/9 follows an ordered sequential Bi Bi mechanism (Fig. 4). Similarly, the histone H3K4 methyltransferase MLL4 (25). and protein arginine methyltransferases (PRMT) 1 and 6 must bind SAM before the protein substrate, therefore these enzymes also follow an ordered sequential Bi Bi mechanism (26, 27, 28, 29).

For many SAM-dependent protein methyltransferases, the SAM/SAH binding site has a higher affinity than the methyl acceptor binding site. The histone H4K20 mono-methyltransferase SET8 has a SAM K_M_ of 16 μM and a histone H4 K_M_ of 40 μM (30). Similarly, the SAM K_M_ values for PRMT 1 and 6 are lower than the K_M_ values for their respective protein substrates (26, 27). In comparison, SET7/9 has a relatively weak SAM/SAH binding site as measured by the SAH K_i_ and SAM K_S_ and K_M_, whereas the histone H3 K_M_ is 8.8-fold lower (Table 1). Such weak SAM/SAH binding may suggest that SET7/9 has relative insensitivity to high intracellular SAH concentrations and sensitivity to low SAM concentrations (31).

### Stability of histone octamers

Histone octamers are most stable at high ionic strength (*e.g.*, ∼0.8–2 M NaCl) and high pH, whereas NCPs are most stable at lower ionic strength, 0.1 to 0.4 M NaCl (32, 33). To make our results comparable, we chose a single condition (buffer, pH, temperature, *etc.*) to perform all experiments with free histones, histone octamers, NCP, and Ub-NCP. We also had to consider the potential for high salt concentrations to suppress signal with analytical mass spectrometry and proteomic experiments (a phenomenon known as “ion suppression”), and the pH maximum for SET7/9 activity (Fig. S3). We therefore used a buffer with 100 mM (NH_4_)HCO_3_ at pH 9. Moreover, given the difficulty we had getting useful data and broad sequence coverage with our proteomics experiments with free histones, we used histone octamers in place of NCP because they lack DNA, which might have interfered with the proteomics experiments. We also had a greater quantity of histone octamers, which we found that we needed for proteomics experiments.

Histone octamers exist in an equilibrium with a histone H3 and H4 tetramer ((H3-H4)_2_) and two histone H2A, H2B dimers (2(H2A-H2B)) (32). Salt concentrations between ∼0.8 to 2 M NaCl displace this equilibrium to the octamer, whereas concentrations lower than 0.6 M NaCl displace the equilibrium to (H3-H4)_2_ + 2(H2A-H2B) (32, 33). In addition, 10.5 > pH < 5 displaces the equilibrium to (H3-H4)_2_ + 2(H2A-H2B) but 7> pH < 9.5 promotes the formation of the octamer, with pH 9.5 yielding maximum octamer stability (32). Given our conditions of low salt and pH 9, the histone octamers in our experiments likely exist as an equilibrium of at least equal parts octamers + (H3-H4)_2_ + 2(H2A-H2B). This is corroborated by the similar sigmoidal rate versus substrate curves and comparable K^'^_B_, V_Max App,_ and specificity constants (k_cat_/K^'^_B_)_App_ with NCP and histone octamers (Fig. 6 and Table 3). However, it is important to note that the sites and types of methylation we identified on histone octamers (Fig. 7*B*) may not be the same as those in NCP.

For the experiments involving free histones, the sequences were derived from *Xenopus laevis,* whereas for histone octamers, NCP, and Ub-NCP experiments, the histones had the human sequence. Human histones H2A, H2B, H3.1, and H4 have 93.8%, 93.4%, 98.5% and 100% residue identity*,* respectively, with the corresponding histone sequences in *Xenopus laevis.* Moreover, none of the minor sequence differences involves lysine or arginine residues that we identified as being methylated. Although we cannot rule out these variations as causing any of the differences we observed between free histones and histone octamers, NCP, or Ub-NCP, we think that this is unlikely given their nearly identical sequences.

### Sigmoidal dependence of rate on NCP, Ub-NCP, or histone octamers

A study by Chuikov *et al.* failed to detect SET7/9 catalyzed NCP methylation, leading to the assumption that SET7/9 cannot methylate nucleosomes *in vivo* (21). Since this study, many have repeated this claim without further experimentation (20, 22, 23, 24). However, we show that SET7/9 can methylate NCP and Ub-NCP (Fig. 6). Care must be taken in the interpretation of the kinetic parameters in Table 3 based on Figure 6. Since we could not saturate SET7/9 with either histone octamers, NCP, or Ub-NCP, the determination of V_Max_ _App_ using Equation 7 is based on the non-linear least squares fitting. K^'^_B App_ and K_M App_ are not equivalent but as others have done, we have calculated an apparent (k_cat_/K^'^_B_)_App_ as a surrogate of the apparent specificity constant (k_cat_/K_M_)_App_ (34, 35, 36, 37). With these caveats in mind, we note that the V_Max_ _App_ for NCPs is at least 9-fold greater, and the V_Max App_ for Ub-NCP is at least 1.8-fold higher than the V_Max App_ for any of the free histones. The specificity constant (k_cat_/K^'^_B_)_App_ for NCP and octamers is 11-; 6.4; 4.0-; and 45-fold higher than the (k_cat_/K_M_)_App_ values for histones H3, H2A, H2B, or H4, respectively. Combined with the sigmoidal curves showing allostery, this suggests not only that SET7/9 catalyzed methylation of NCP is possible *in vivo* but that nucleosomes (with and without Ub) are the preferred substrates.

Depending on their value, Hill coefficients can suggest the presence of an allosteric interaction. However, Hill coefficients cannot be related to a specific number of binding sites but are only a rough indicator of an allosteric interaction where n_H_ > 1 is indicative of a positive allosteric interaction, n_H_ = 0 indicates no allostery, and n_H_ < 1 indicates a negative allosteric interaction. The Hill coefficients (n_H_) for NCP, Ub-NCP, and histone octamers are 2.062, 2.418, and 1.748, respectively. Therefore, these Hill coefficients suggest a positive allosteric interaction that is only observed between SET7/9 and NCPs (with or without Ub) or octamers. Moreover, the similarity in (k_cat_/K^'^_B_)_App_ for NCP and histone octamers suggests that the DNA in the NCPs is not involved in this interaction (Table 3).

The sigmoidal rate vs substrate curves might be explained by cooperativity between sites in a random sequential Bi-Bi mechanism or a multimeric enzyme. However, this cannot explain the behavior of SET7/9, as it exhibits an ordered sequential Bi-Bi mechanism, and to the best of our knowledge, SET7/9 exists as a monomer. Furthermore, we cannot attribute this activity to the presence of multiple sites of methylation, as sigmoidal curves were not observed with the free histones, where we also observed multiple sites of methylation.

Sigmoidal rate vs substrate curves have been observed with monomeric enzymes where a slow conformation change occurs, such as with hexokinase (38) and the histone acetyl-transferases p300 and CBP (34, 35). This mechanism assumes a random ordered Bi-Bi enzymatic mechanism manifesting as sigmoidal substrate versus rate curves with one substrate fixed and the other varied, and hyperbolic curves when the fixed and varied substrates are reversed (38). We did not find a random ordered Bi Bi mechanism for SET7/9, nor did we observe sigmoidal curves in our kinetic analysis when histone H3 was fixed and SAM varied, nor when SAM was fixed and histone H3 varied, nor with changing concentrations of any of the free histones in the presence of fixed saturating SAM. Accordingly, we rule out the slow conformation change mechanism as explaining the apparent allosteric activity of SET7/9 with histone octamers, NCP, and Ub-NCP. Instead, our data suggest that there is an allosteric site on SET7/9 that binds to one histone while the target lysine of another histone that is part of an octamer, NCP, or UbNCP is in the active site. A possible candidate for this allosteric site is the three MORN domains of SET7/9, which bind positively charged amino acids (39). In fact, the MORN domains of SET7/9 were shown to bind to a sequence from K14 to H39 on a peptide of the N-terminus of histone H3, where the interaction positions SET7/9 for methylation of histone H3K4 following hyperbolic kinetics (40). We speculate that, in the context of the histone octamer, NCP or Ub-NCP, binding of the SET7/9 MORN domains to the histone H3 N-terminal tail results in an allosteric interaction and methylation of neighboring histones following sigmoidal kinetics. The implications of this will be discussed in more depth below.

### Methylation sites on free histones and histone octamers

We determined specific sites where SET7/9 can methylate free histones and histone octamers. Our study differs from others since we used full-length histones and octamers and proteomic analysis rather than N-terminal tail peptides and scanning alanine mutagenesis (9, 10). We confirmed that SET7/9 produces histone H2BK15me1(9) but found that it also produced histone H2BK11me1, H2BK12me1, H2BK15me1, H2BK16me1, and H2BK20me1/me2 in the free histone H2B tail, H2BK46me1 in the core, and H2BK116me1 and H2BK120me1 in the C-terminus. In the context of an octamer, slightly different histone H2B methylation was observed, H2BK5me1/me2, and dimethylation of H2BK11, 12, 15, and 16, but a lack of H2BK20me2. The role of this histone H2B N-terminal tail methylation is unclear, but CPB/p300-catalyzed acetylation of histone H2BK5, 12, 15, and 20 (41, 42) is referred to as histone H2B N-terminus multisite lysine acetylation and marks active enhancers (42). Such acetylation would be mutually exclusive with SET7/9 catalyzed methylation at the same sites. Interestingly, histone H2BK20ac has been identified as the “most predictive mark of active enhancers” and, in some cases, was considered more important than histone H3K27ac (43). Thus, histone H2BN-terminal tail methylation by SET7/9 may prevent H2B N-terminus multisite lysine acetylation, reducing the activity of active enhancers.

Histone H3 has the highest number of sites of methylation of all free histones. Every K and R residue in the first 42 residues of histone H3 is at least mono-methylated. In contrast, we detected no sites of methylation in the first 17 residues of histone H3 within an octamer, only detecting histone H3K18me1, H3K36me1, H3K56me1, and H3K122me1. The lack of histone H3K4me1 in the octamer may explain why others have failed to detect SET7/9 catalyzed methylation of NCP, since this specific site is assumed to be the only or most important methylation site for SET7/9 (20, 22, 23, 24). Therefore, if one was using Western blotting for SET7/9 catalyzed histone H3K4me1 on NCP, our results suggest that no histone H3K4me1 would be detected. The difference in methylation between free histone H3 and histone H3 in an octamer may be mediated by the MORN domains of SET7/9. The MORN domains may bind to the solvent-exposed histone H3 N-terminal tail in a nucleosome in an orientation that positions the catalytic domain to methylate the C-terminus of histone H3 and neighboring histones but not histone H3K4. This might explain why there is no methylation in the first 17 residues of histone H3 when it is part of an octamer. Moreover, our results confirm that SET7/9 catalyzed histone H3K4me1 can happen prior to nucleosome assembly but not after (7, 8).

Many of the SET7/9 methylation sites we identified on histones are known sites of ubiquitination, including histone H2AK129, H2BK120, and H3K122. We found histone H2AK129me1 in free histone H2A, but in the octamer, our coverage did not include this residue, so it is unclear if this modification exists in the octamer. Interestingly, histone H2AK129 is part of a R/KSK motif, which is a methylation consensus sequence for SET7/9 (9). Histone H2A has several sites of mono-ubiquitination, but relevant to this discussion, BRCA1/BARD1 mono-ubiquitinates histone H2AK127 and H2AK129 (44). Since lysine methylation and ubiquitination are mutually exclusive, our results suggest that SET7/9 may methylate histone H2AK129, preventing subsequent mono-ubiquitination at that site (45).

H2BK120Ub is found in actively transcribed genes (39) and induces changes in nucleosome structure that result in increased active marks, histone H3K79 and H3K4 mono- and di-methylation catalyzed by DOT1L (46) and MLL1 (47), respectively. Histone H3K4 methylation catalyzed by MLL1 or its yeast cognate Set1 appears to be dependent on prior H2BK120Ub (47, 48). H2BK120Ub prevents chromatin compaction, yielding open chromatin, increasing the interaction between MLL1 and nucleosomes, and increasing histone H3K4 methylation (47). SET7/9 catalyzed H2BK120me1 may block subsequent H2BK120Ub, leading to reduced MLL1-mediated H3K4me1.

Active enhancers can be superficially characterized on a histone level by the presence of histone H3K4me1 and H3K27ac or H2BK20ac. Previously, SET7/9 was thought to play a role in constructing active enhancers by catalyzing histone H3K4me1, prior to nucleosome assembly (8). Our results confirm the formation of histone H3K4me1 only on free histone H3, but we also reveal many other sites and types of methylation on free histones and nucleosomes. In the context of the nucleosome, we show that the net effect of SET7/9 may be to reduce the activity of active enhancers by, not catalyzing H3K4me1, by preventing histone H2B N-terminus multisite lysine acetylation, including H2B20ac, and by blocking H2BK120Ub, thereby leading to reduced MLL1-mediated H3K4me1.

The ubiquitination of histone H2BK120, in Ub-NCP blocks subsequent methylation at that site, and this could explain why the Ub-NCP V_max_ _App_ is ⅕ of that for NCP. Nevertheless, SET7/9 exhibits sigmoidal kinetics with Ub-NCP and, unlike any other substrate, the production of Kme1 and Kme2 at equal rates (Fig. 6, *B* and *C*) with low enzyme and short incubation times, suggesting it has *in vivo* relevance. It was previously thought that wild-type SET7/9 only catalyzes the formation of Kme1, and initial structural studies suggested that SET7/9 cannot catalyze the formation of Kme2 or Kme3 because of restricted volume in the active site (12). As noted, mutations in the active site may increase this volume, allowing the formation of Kme2 and limited Kme3 (6, 12). Absent such a mutation, it's difficult to understand how Ub-NCP can yield Kme2. However, structural studies with MLL1, MLL3, or DOT1L bound to Ub-NCP show structural changes in the enzyme that permit higher methylation activity on Ub-NCP and cause distinct changes in binding compared to NCP (46, 47). Similar structural changes in binding between SET7/9 and Ub-NCP may alter active site architecture to allow formation of Kme1 and Kme2 without the need for the high SET7/9 and SAM and extended incubation times needed to produce Kme2 and Kme3 in Figure 7, *A* and *B*, *D* and *E*.

Most surprising is the finding of SET7/9 dependent formation of Rme1 and Rme2a (known as Type I arginine methylation). This is the first finding that Rme1 and Rme2a formation can be catalyzed by any enzyme other than PRMTs. Given the exhaustive methylation conditions needed to observe this, the biological relevance of Rme1 and Rme2a formation by SET7/9 is unclear. However, recent selectivity studies with inhibitors of PRMT1 (a Type I PRMT) show that some PRMT1 inhibitors are also low-potency SET7/9 inhibitors (49, 50) suggesting that there is sufficient similarity between the active sites of SET7/9 and PRMT1 that allows these inhibitors to bind to both enzymes (51).

## Experimental procedures

### Protein expression and purification

The constructs for *Xenopus laevis* histones H2A, H2B, H3, and H4 in the pET3a vector were a generous gift from Dr Karolin Luger at the University of Colorado, which were transformed into BL21(DE3) pLysS *E coli* cells, grown, and induced according to previous methods (52). Inclusion bodies were prepared from the pellets and histones denatured, renatured, and purified according to previous methods (53) (see supplemental methods for additional details). Full-length human SET7/9 in the pET28a-LIC vector was a generous gift from the laboratory of Dr Masoud Vedadi of the Ontario Institute of Cancer Research. Details on the expression of SET7/9 can be found in the supplemental methods.

### Methylation reactions

Methylation reactions contained SET7/9, free *Xenopus laevis* histones, human recombinant histone octamers (EpiCypher EP16–0001), human recombinant NCP (EpiCypher EP160009), or human recombinant Ub-NCP (EpiCypher EP160396) and SAM (Cayman Chemical Co.) in a buffer with 100 mM (NH_4_)HCO_3_ pH9, and 200 nM internal standard stable isotope labeled ^13^C6 ^15^N2 lysine, at 30 °C. The NCP, UbNCP, and octamers have been used by many groups, having been validated by SDS-PAGE, mass spectrometry, and Western blot previously (54, 55, 56). The details for the various methylation reactions are in the supplemental methods. Dried samples were acid hydrolysed in the vapor phase using 6M HCl at 110 °C for 24 h, and the hydrolyzed samples were dried in a vacuum centrifuge and reconstituted in 100 μl of 0.05% formic acid for analysis by LC-MS/MS (see below). The concentration of free histones, histone octamers, NCP, Ub-NCP, and SET7/9 was determined by acid hydrolysis and quantification of K and R using LC-MS/MS (below). SAM stock concentrations were determined by UV absorbance and the molar extinction coefficient 15,400 L mol^−1^ cm^−1^ at 260 nm (57). Details on the methylation reactions can be found in the supplemental materials.

### Analytical LC-MS/MS

We previously developed and methods to quantify multiple post-translational modifications including lysine (K) acetyl-lysine (Kac), mono- (Kme1) di- (Kme2) and trimethyllysine (Kme3), arginine (R) monomethylarginine (Rme1), asymmetric-dimethylarginine (Rme2a), and symmetric-dimethylarginine (Rme2s) that were validated using full length histones with a single site of modification (13, 14, 15). This method was modified to quantify methylated lysines and arginines with the internal standard ^13^C6 ^15^N2 lysine for enzymatic reactions. Hydrolyzed samples were analysed using a Nexera UHPLC connected to a Shimadzu 8040 triple quadrupole mass spectrometry system. Chromatographic separation was achieved using a Primesep200 (SIELC) 4.6 x 150 mm 5 μm 100 Å HPLC column at 40 °C. Mobile phases (A) 0.05% formic acid in water and (B) 1% formic acid in 50% aqueous acetonitrile were used at a flow rate of 0.4 ml/min. Initial conditions were 0% (B) for 1.5 min, increasing to 85% (B) over 30 s and held for 3 min. Positive ion analytes were quantified using multiple reaction monitoring (MRM) and dual ESI, APCI (DUIS) ionization. The precursor and product ion *m/z* values collision energies and other parameters are in Table S1. The nebulizing gas was set to 2 L/min, drying gas was 15 L/min, desolvation line temperature was 250 °C and the heating block 400 °C. The assay can quantify K, Kme1, Kme2, Kme3, R, Rme1, Rme2a, and Rme2s.

### Data analysis

Enzymatic rate data and substrate histone H3 and SAM concentrations for the kinetic analysis of SET7/9 and product inhibitor studies were reciprocal transformed and fit to a straight-line equation with 1/Y^2^ weighting (to account for bias due to reciprocal transformation) using SigmaPlot 14.5. The hyperbolic rate data for the activity of SET7/9 against free histones H2A, H2B, H3, and H4 were fit to the Michaelis–Menten equation (Eq. 5 and 6), and the sigmoidal rate data for SET7/9 activity on NCP with and without histone H2BK120 ubiquitination and histone octamers were fit to the Hill equation (Eq. 7) using SigmaPlot 14.5.

### Proteomic experiments

Extended incubation experiments were performed with 0.5 μM SET7/9, 100 mM (NH_4_)HCO_3_ buffer (pH9), 200 nM internal standard ^13^C6 ^15^N2 lysine, 10 μM H2A, H2B, H3, H4 or 1 μM histone octamer substrate, with and without 500 μM SAM. The reactions were carried out at 30 °C with an incubation time of 24 h.

Per sample, 3 pmol were incubated with 10 mM dithiothreitol to reduce disulfide bonds, and free cysteines were alkylated with 10 mM iodoacetamide for 30 min at RT in the dark. Samples were digested by adding the broad specificity protease subtilisin (Sigma Aldrich) at a 1:10 ratio (protease: protein) for 20 min at 56 °C (58). The digestion was quenched by the addition of 1% TFA, and 500 fmol per digest were analyzed by nanoflow LC-MS/MS using an Easy-nLC 1200 system coupled to an Exploris 480 (Thermo Fisher Scientific).

Per samples, 500 fmol aliquots were separated on a self-packed C18 analytical column (Luna C18(2), 3 μm particle, 100 μm ID x 30 cm, Phenomenex, Torrance, CA) using water with 0.1% FA (mobile phase A) and 80% ACN with 0.1% FA (mobile phase B) using a binary gradient from 2 to 6% B over 3 min, 6 to 30% B over 36 min, 30 to 50% B over 5 min, 50 to 100% B over 1 min at a flow rate of 300 nl/min. MS acquisition was conducted in data-dependent acquisition mode (DDA) with a top 10 method. Full MS scans were acquired at a resolution of 45,000 from 380 to 1500 m/z (AGC 300%, 45 ms max injection time). Precursor ions with charge states between +2 and +5 were isolated with a *m/z* 1.6 window and fragmented with a normalized collision energy of 30%. MS/MS spectra were acquired at a resolution of 45,000 (AGC 100%, max injection time set to auto). Dynamic exclusion was set to 5 s. For free histone samples, Proteome Discoverer 3.01.27 was used for data analysis with unspecific digestion, 10 ppm was allowed a maximum precursor mass deviation, and 0.02 Da for fragment ions. A subset of the human proteome database with 6516 protein entries without histone sequences was used, and the *Xenopus* histone sequences were appended for free histones. Mono-, di-, and tri-methylation of Lys and Arg, as well as oxidation of Met residues, were allowed as variable and carbamidomethylation of Cys was set as a fixed modification. All matched peptide sequences passing a 5% false discovery rate (target/decoy approach with Percolator) and matching only the expected histone protein (uniqueness) were considered for data analysis. Only lysine and arginine modifications that could be localized with >90% site probability to a specific Lys residue were considered. Peptide intensities (area under the curve) were determined using label-free quantitation (LFQ) and compared between +SAM and −SAM samples, where required, these were normalized to reflect equal overall histone target intensities. A complete list of peptide intensities for the free histone samples is in Sheet S1.

Octamer samples were searched using FragPipe 23.0 and a human Swissprot database (downloaded April 2025). To avoid ambiguous assignment of octamer-histone peptides, all histone sequences other than human histone H2A (H2AC4 (P04908)), histone H2B (H2BC12 (O60814)), histone H3.1 (H3C1 (P68431)) and histone H4 (H4C1 (P62805)) were removed, resulting in a total of 20,383 target sequences, including common contaminants. Nonspecific cleavage was selected, peptide length was set from 7 to 25 amino acids, precursor and product ion mass tolerances were set to 20 ppm. Oxidation of Met, as well as mono-, di-, and tri-methylation of Lys residues were allowed as variable modifications. These settings resulted in a total of 1,677,783 modified peptides considered in the search. As above, because of the low probability of having random peptide-spectrum-matches to the four octamer proteins, a false-discovery-rate of 5% was allowed on the peptide level. LFQ was performed using default IonQuant settings including the default minimum site-localization probability of 0.75. A complete list of peptide intensities for the octamers are in Sheet S2.

## Data availability

Source data are provided with this paper.

## Supporting information

This article contains supporting information.

## Conflict of interest

The authors declare that they have no conflicts of interest with the contents of this article.

## Supporting information

Supplemental file

Supplemental Sheet 1

Supplemental Sheet 2

Data Ige SET79 20250722
