# Supplementary material for: SET7/9 exhibits sigmoidal kinetics on nucleosomes, hyperbolic kinetics on histones by an ordered sequential mechanism, and methylates lysine and arginine
Source: J Biol Chem. 2025 Aug 28;301(10):110639. doi: 10.1016/j.jbc.2025.110639 (PMC12514578; doi:10.1016/j.jbc.2025.110639)
Supplement: Supplemental file [file mmc1.docx]

Supporting Information for

SET7/9 exhibits sigmoidal kinetics on nucleosomes, hyperbolic kinetics on histones by an ordered sequential mechanism and methylates lysine and arginine

Olufola O. Ige^1,2^, Thordur Hendrickson-Rebizant^1,2^, Wenxia Luo^1,2^, Phinehas Cheung^1^, Ying Lao^3,4^, Rene P. Zahedi^2,3,4,5^, James R. Davie^2,5^, Ted M. Lakowski^1,2*^.

^1^Pharmaceutical analysis Laboratory, College of Pharmacy, University of Manitoba, 750 McDermot Avenue West, Winnipeg, Manitoba R3E 0T5, Canada

^2^Paul Albrechtsen Research Institute, CancerCare Manitoba, Winnipeg, MB R3E 0V9, Canada

^3^Manitoba Centre for Proteomics and Systems Biology Winnipeg, Manitoba R3E 3P4

^4^Department of Internal Medicine Winnipeg, Manitoba R3A 1R9

^5^Department of Biochemistry and Medical Genetics, University of Manitoba, Winnipeg, MB R3E 0J9, Canada

^*^Address correspondence to Ted Lakowski University of Manitoba ted.lakowski@umanitoba.ca

**Supplemental methods**

***Histones expression and purification of Histones***

E. coli BL21(DE3) pLysS cells containing the genes for Xenopus Laevis histones H2A, H2B, H3 and H4 in the pET3a vector were a generous gift from Dr Karolin Luger at the University of Colorado that were transformed into E. coli BL21(DE3) pLysS E coli, grown, and induced according to previous methods Briefly, bacteria were cultured in 2x LB both at 37°C and induced with 0.2mM IPTG for 2 h, then centrifuged at 15000 x g for 20 minutes at 4°C. The pellet was suspended in a lysis buffer (50 mM Tris pH 7.5, 100 mM NaCl, 1 mM PMSF, 1 mM BME, 2 protease inhibitor tablets (Life Technologies), 20 U DNAse, 0.33 mg/mL lysozyme (Amresco)) and sonicated using a Branson Sonifier with 30s and breaks on ice. Inclusion bodies were prepared from the pellets and histones denatured, renatured, and purified according to previous methods. The resulting suspension was washed with lysis buffer but with the addition of 1% Triton X, sonicated centrifuged at 4°C at 12,000 x g for 20 minutes and the supernatant is decanted. The pellets wash steps were repeated at least 3 times until inclusion bodies were obtained. The inclusion bodies were denatured in a buffer containing 20 mM Tris, 10M guanidine hydrochloride and 10mM DTT for 1hr and centrifuged at 1200 x g to separate the pellets. The inclusion bodies refolded in a buffer containing 100 mM Tris, 400 mM arginine, 2mM EDTA, 2mM DTT and 2mM PMSF. The solution containing the denatured buffer was added at around 1mL/min on ice. The resulting solution is concentrated with Amicon® Ultra Centrifugal Filter, 10 kDa MWCO at 4000 x g for 45 minutes. The concentrate was collected and injected into a Shimadzu 10A VP series HPLC. Mobile phase A is 0.1% Trifluoroacetic acid in H2O, B is 0.1% Trifluoroacetic acid in acetonitrile at 2mL/min. Initial conditions were 5% (B) for 5 minutes, increasing to 65% (B) over 28 minutes and then increased to 90% (B) for 2 minutes and held for 3 minutes. The run was monitored at 260 and 280nM. The column was a semi preparative ZORBAX C8 9.4x250mm. The peaks were collected, analyzed with Protein SDS Gel electrophoresis and freeze dried with a Labconco FreeZone 4.5 Liter Benchtop Freeze Dry System and then stored at -80°C.

***SET7/9 expression and purification***

Full-length human SET7/9 in the pET28a-LIC vector was a generous gift from the laboratory of Dr. Masoud Vedadi of the Ontario Institute of Cancer Research. SET7/9 was expressed in BL21(DE3) pLysS cells (Invitrogen) induced with 1 mM IPTG and incubated at ambient temperature (~27°C) with shaking at 300 rpm overnight (16 h), centrifuged at 15000 x g for 20 minutes at 4°C. The pellet was resuspended in 20 mM NaHPO4, (pH7.5) 100 mM NaCl, 1 mM PMSF, 7 mM β-mercaptoethanol, 0.1% Triton X-100, 0.5 mg/mL lysozyme with an EDTA-free protease inhibitor tablet (Life Technologies) and 10 U DNase, and sonicated on ice using a Branson Sonifier, centrifuged and the supernatant loaded onto a 5 mL HisTrap FF column (GE Healthcare) connected to an AKTÄ Purifier FPLC at a flow rate of 1 mL/min. SET7/9 was purified with a step gradient of A: 20 mM NaHPO_4_, pH7.5 100 mM NaCl, 1 mM PMSF, 7 mM β-mercaptoethanol and B: 20 mM NaHPO_4_, pH7.5 100 mM NaCl, 1 mM PMSF, 7 mM β-mercaptoethanol with 250 mM imidazole. The column was washed with 70mL of A, then 10 mL of 10% B, then SET7/9 eluted with 15 mL of 100% B, all flow rates were 3 mL/min. The eluted fraction was concentrated with a Spin-X UF20 concentrator 10 kDa NMWCO concentrator (Corning) and dissolved in a storage buffer with 50mM Tris pH7.5 with 5% glycerol and re-concentrated.

***Details of methylation reactions***

To determine where the reaction rate (in pmol/min) for SET7/9, SAM and histone H3 is increasing linearly over time, SET7/9 (100nM) was incubated with high and low concentrations of substrates SAM (25 µM and 5 µM) and histone H3 (5 µM and 1 µM). The samples were analyzed at 5, 10, 20, 30, 40 60, 120, 240 and 360 minutes in a buffer with (NH_4_)HCO_3_ buffer (pH9) (Sigma-Aldrich)(100 mM), internal standard stable isotope labeled ^13^C6 ^15^N2 lysine (Cambridge Isotopes Laboratories Inc.) (200 nM) (S. Fig. 1).

**S. Fig. 1.**

Time course activity of high and low concentrations of SAM (25 µM and 5 µM) and histone H3 (5 µM and 1 µM) with 100 nM of SET 7/9. This is used to determine where the reaction rate for SET7/9, SAM and histone H3 is increasing linearly over time. The Mass Spectrometry parameters are listed in Table S1.

**Table S1.** Mass Spectrometry parameters: MRM transitions and collision energies.

| **Analyte** | **Retention Time**  **(min.)** | **Precursor>product ion**  **(*m/z*)** | **Collision Energy**  **(eV)** |
| --- | --- | --- | --- |
| K | 2.03 | 146.9>130.0 | -14 |
| Kme1 | 3.34 | 161.2>84.0 | -18 |
| Kme2 | 3.42 | 174.8>84.0 | -20 |
| Kme3 | 3.5 | 189.2>83.9 | -22 |
| R | 2.15 | 175.2>70.10 | -14 |
| Rme1 | 3.41 | 188.8>70.0 | -24 |
| Rme2s | 3.46 | 202.8>171.9 | -14 |
| Rme2a | 3.46 | 203.0>45.9 | -14 |
| ^13^C_6_ ^15^N_2_ lysine | 2.07 | 155.0>90.2 | -17 |

To determine the range over which the rate of reaction for SET7/9 with SAM and histone H3 increases linearly with enzyme concentration, SET7/9 was incubated with SAM and H3 (1 with 3.125, 6.25, 12.5, 25, 50, 100 and 200nM SET7/9 (S. Fig. 2).

**S. Fig. 2.**

The activity of increasing concentrations of SET 7/9 with 5 µM SAM and 1 µM histone H3. This shows the rate of reaction for SET7/9 with SAM and histone H3 increases linearly with enzyme concentration up to at least 200 nM.

Effect of pH on the activity of SET7/9 was determined with NH_4_)HCO_3_ buffer (100 mM) varying from pH6 to pH10 with 5 µM SAM and 1 µM histone H3 . The samples were stoped at 30 minutes (S. Fig. 3).

**S. Fig. 3.**

The activity of SET 7/9 at various pH points. SET 7/9 shows its highest activity at pH 9. Each bar is the mean and S.D. of 2 repeats.

The effect of DTT on the enzymatic activity of SET7/9 was determined by incubating the enzyme in one group with SAM histone H3 (5 µM and 1 µM respectively) and (NH_4_)HCO_3_ buffer (pH9) (100 mM), internal standard stable isotope labeled ^13^C6 ^15^N2 lysine (200 nM) and 2.5 mM DTT and another group that did not contain DTT. The samples were analyzed at 15, 30 and 60 minutes (S. Fig. 4).

Given the results from these experiments, all subsequent kinetic methylation reactions were carried out for 20min with 25nM SET7/9 in a buffer with 100 mM (NH_4_)HCO_3_ at pH 9, and 200 nM ^13^C6 ^15^N2 lysine internal standard, unless otherwise stated.

**S. Fig. 4.**

The activity of SET 7/9 with and without DTT. Analysis using paired t-test shows the differences due to the presence of DTT are not statistically significant (p>0.05) up to 60 mins.

***Substrate control and SET7/9 auto-methylation experiments***

To assess the potential auto-methylation activity of SET7/9, enzymatic reactions were performed under different conditions in the presence and absence of histone H3 and SAM. The reactions included a complete reaction containing 30 µM histone H3, 50 nM SET7/9, and 100 µM SAM; a control reaction with only SET7/9 and SAM (50 nM SET7/9 and 100 µM SAM); another control with only SET7/9 and histone H3 (30 µM histone H3 and 50 nM SET7/9); and a final control with histone H3 and SAM only (30 µM histone H3 and 100 µM SAM). Following a 30-minute incubation, the samples were analyzed to evaluate auto-methylation. The reactions were carried out in 100 mM (NH_4_)HCO_3_ pH9 () 200 nM ^13^C6 ^15^N2 lysine (S. Fig. 5**)**

**S. Fig. 5.**

Substrate control assay for SET7/9. To determine whether SET7/9 exhibits auto-methylation. Enzymatic reactions were conducted in the absence of histone H3 and/or SAM. The methylation signals detected under these conditions were below the limit of quantification, indicating that SET7/9 does not undergo auto-methylation. These results confirm that the methylation activity of SET7/9 is strictly dependent on the presence of a viable substrate and SAM. Each bar is the mean and S.D. of 2 repeats. # indicates the methylation signals detected under these conditions were below the limit of detection. * indicates the methylation signals detected under these conditions were below the limit of quantification.

***Preliminary product inhibitor experiments***

*Xenopus laevis* histone H3Kme1 methylated lysine analog (MLA) (H3K4me1MLA) was purchased from ActiveMotif (31719) and SAH >98% from Cayman.

Preliminary measurements of the inhibition of SET7/9 by histone H3K4me1MLA were determined by incubating 25nM SET7/9, 20 µM SAM , 6 µM histone H3 and histone H3K4me1MLA at concentrations of 1.56, 3.125, 6.25, 12.5, 25, 50 and 100 µM (S. Fig. 7).

**S. Fig. 6.**

IC_50_ curve of the inhibition of SET7/9 by H3K4me1MLA with 25 nM of SET 7/9 5 µM SAM and 1 µM histone H3 . The IC_50_ value for histone H3K4me1MLA is 15.485 µM.

Preliminary measurements of the inhibition of SET7/9 by SAH were determined by incubating 20 µM SAM, 6 µM histone H3 and SAH at concentrations of 0.001, 0.01, 0.1, 1, 10, 100, 1000 µM (S. Fig. 8).

**S. Fig. 7.**

IC_50_ curve of the inhibition of SET7/9 by SAH. The IC_50_ value for SAH is 13.081 µM.

***Determination of V_Max_ and K_M_***

The experiments to determine V_Max_ and K_M_ values for histone H3 and SAM were as follows 0.47, 0.94, 1.88, 3.75, 15 and 30 µM µM of histone H3 were incubated with SAM concentrations of 6.25, 12.5, 25, 50, 100 and 200 µM and for a total of 36 samples. Replots from Figure 2 (S. Fig. 6) are shown for the slopes from the reciprocal V_o_ vs reciprocal histone H3 plotted against reciprocal SAM, the reciprocal intercept (V_Max App_) for the plots of reciprocal V_o_ vs reciprocal histone H3, plotted against reciprocal SAM, and the reciprocal intercept (V_Max App_) for the plots of reciprocal Vo vs reciprocal SAM, plotted against reciprocal histone H3.


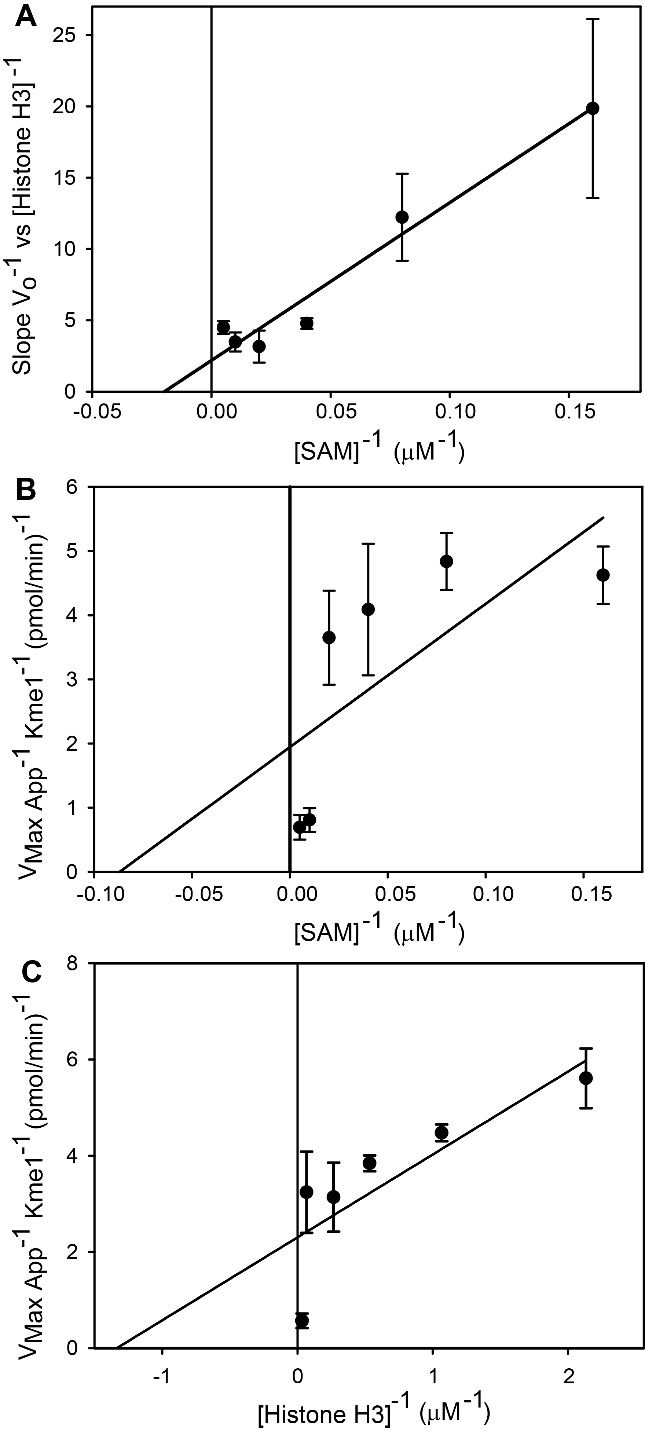


**S. Fig. 8.**

Replots from Figure 2 for the slopes from the reciprocal V_o_ vs reciprocal histone H3 plotted against reciprocal SAM (A) the reciprocal intercept (V_Max App_) for the plots of reciprocal V_o_ vs reciprocal histone H3, plotted against reciprocal SAM (B), and the reciprocal intercept (V_Max App_) for the plots of reciprocal Vo vs reciprocal SAM, plotted against reciprocal histone H3 (C).

***Product inhibitor experiments***

The product inhibitor experiments for SAH against histone H3 (Fig. 3A) were performed with a fixed 30 µM of SAM with 0.625, 1.25, 2.5, 5, 10 and 20 µM histone H3 and 12.5, 25, 50, 100 and 200 µM of SAH. 4 samples were not plotted at the highest concentrations of SAH and lowest of histone H3: 200 SAH and 0.625, 1.25 and 2.5 µM histone H3 and 100µM of SAH and 0.625 µM histone H3 because the measured Kme1 was below the calibrated range or LLOQ of our assay.

The product inhibitor experiments for SAH against SAM (Fig.3B) were performed with fixed concentrations of 4µM of histone H3 with 3.125, 6.25, 12.5, 25, 50, 100 and 200 µM of SAM and 6.25, 12.5, 25, 50, 100 and 200 µM of SAH.

The product inhibitor experiments for histone H3K4me1MLA against histone H3 (Fig. 3C) were performed with a fixed 30µM of SAM with and 0.625, 1.25, 2.5, 5, 10 and 20µM of histone H3 and 15.6, 31.2, 62.5, 125, 250 µM of histone H3K4me1MLA.

The product inhibitor experiments for histone H3K4me1MLA against SAM (Fig. 3D) were performed with fixed 4 µM histone H3 with 6.25, 12.5, 25, 50, 100 and 200 µM of SAM and 7.8125, 31.25, 62.5, 125, 250 µM of histone H3K4me1MLA. The replots from Fig 3 are shown in S. Fig. 9.


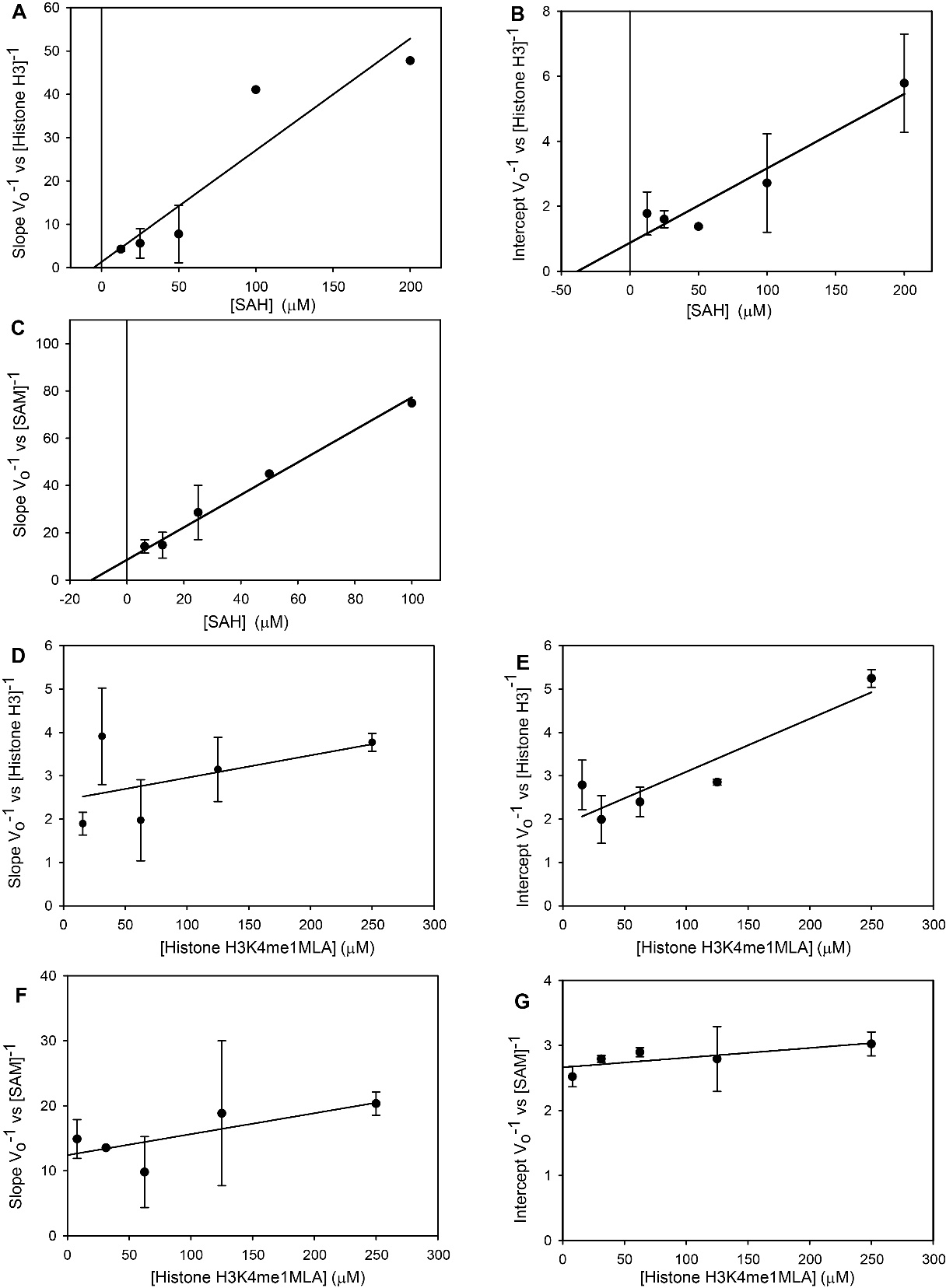


**Fig. S9.**

The replots from Fig 3 for slope (A) and intercept (B) where histone H3 is the substrate and SAH is the product inhibitor, slope (C) where SAM is the substrate and SAH the product inhibitor. The replots for slope (D) and intercept (E) where histone H3 is the substrate and histone H3 H3K4me1MLA is the product inhibitor, the slope (F) and intercept (G) where SAM is the substrate and histone H3K4me1MLA the product inhibitor. The product inhibitor constants are summarized in Table S2.

**Table S2.** Product inhibitor constants for SET7/9.

| **Substrate** | **Product** | **K_i slope_ (µM)** | **K_i Int_ (µM)** |
| --- | --- | --- | --- |
| A (SAM) | P (H3K4me1) | 428.2±207 | 1936±900 |
| B (H3) | P (H3K4me1) | 530.9±287 | 167.2±96.0 |
| A (SAM) | Q (SAH) | 12.14±3.11 | NA*^a^* |
| B (H3) | Q (SAH) | 85.21±99.1 | 50.92±48.8 |

*^a^* Cannot be calculated with a sequential ordered model.

Other than what is explicitly stated above no more than 5 points from each group of 30 samples for each set of product inhibitor experiments were rejected for being lower than the calibrated range, below the LLOQ or deviating too much from linearity. The following equations were used to determine the inhibition constants.

$K_{iSlope}^{P/A}=\frac{K_{iQ}K_{MP}}{K_{MQ}}\left( 1+\frac{K_{MA}[B]}{{K_{SA}K}_{MB}} \right)$ [S.Eq.1]

$K_{i Int.}^{P/A}=\frac{K_{iP}\left( 1+\frac{K_{MB}}{[B]} \right)}{\left( 1+\frac{K_{MQ}K_{MB}K_{iP}}{{K_{iQ}K}_{MP}[B]} \right)}$ [S.Eq.2]

$K_{iSlope}^{P/B}=\frac{K_{iQ}K_{MP}}{K_{MQ}}$ [S.Eq.3]

$K_{i Int.}^{P/B}=K_{iP}\left( 1+\frac{K_{MA}}{[A]} \right)$ [S.Eq.4]

$K_{i Slope.}^{Q/A}=K_{iQ}=\frac{k_{4}}{k_{-4}}$ [S.Eq.5]

$K_{i Slope}^{Q/B}=K_{iQ}\left( 1+\frac{[A]}{K_{SA}} \right)$ [S.Eq.6]

$K_{i Int.}^{Q/B}=K_{iQ}\left( 1+\frac{[A]}{K_{MA}} \right)$ [S.Eq.7]

***Apparent Michelis-Menten values of histone H3 and SAM***

The apparent Michelis-Menten parameters V_Max-App_ and K_M-App_ for histone H3 was determined with 25 nM of SET7/9 incubated with 200 µM of SAM and histone H3 at 0.23,0.47, 0.94, 1.88, 3.75, 7.5, 15 and 30 µM. While the apparent Michelis-Menten parameters V_Max-App_ and K_M-app_ for SAM was determined with 30 µM histone H3 and SAM at 3.125, 6.25, 12.5, 25, 50, 100 and 200 µM.

***Histone and nucleosomal substrate profiling***

The apparent Michelis-Menten parameters V_Max-App_ and K_M-App_ for other substrates were determined with 25 nM SET7/9 with SAM fixed at 200 µM. The concentrations of histone H2A were 0.78, 1.56, 3.125, 6.25, 12.5, 25 µM. The concentrations of histone H2B were 0.78, 1.56, 3.125, 6.25, 12.5 µM. The concentrations for histone H4 were at 0.47, 0.94, 1.88, 3.75, 7.5, 15 and 30 µM. The concentrations of histone octamers were 0.0625, 0.125, 0.25, 0.5, 1, 2, 4 µM. The concentrations of NCP were 0.0625, 0.125, 0.25, 0.5, 1, 2, 4 µM. The concentrations of Ub-NCP were 0.047, 0.094, 0.188, 0.375, 0.75, 1.5, 3 µM. For histone octamers, NCP and Ub-NCP the concentration range was dictated by the maximum concentration supplied.
